# Is It Possible to Change from a Linear to a Circular Economy? An Overview of Opportunities and Barriers for European Small and Medium-Sized Enterprise Companies

**DOI:** 10.3390/ijerph16050851

**Published:** 2019-03-08

**Authors:** Concepción Garcés-Ayerbe, Pilar Rivera-Torres, Inés Suárez-Perales, Dante I. Leyva-de la Hiz

**Affiliations:** 1 Department of Management, University of Zaragoza, 50005 Zaragoza, Spain; cgarces@unizar.es; 2 Department of Marketing, University of Zaragoza, 50005 Zaragoza, Spain; privera@unizar.es; 3 Montpellier Business School, 34080 Montpellier, France; d.leyva@montpellier-bs.com

**Keywords:** circular economy, sustainable development, implementation typology, barriers, 4Rs

## Abstract

The Circular Economy is a paradigm shift attempting to replace the end-of-life concept with reducing, reusing, recycling and recovering materials and to slow down, close and narrow material and power loops. This concept is much discussed in the academic literature, but limited progress has been accomplished so far regarding its empirical analysis. The objective of this work is to study circular economy practices and analyze in depth the circular economy behavior in European firms. We find that firms’ circular economy behavior is a gradual process where measures are implemented gradually, starting with activities involving control measures and ending with putting preventive practices in place. We discovered also that the most proactive companies in implementing circular economy measures generally come across certain common barriers such as administrative processes, regulations and a lack of human resources to perform these practices, while firms that have not implemented circular economy measures view financing, investment and cost–benefit barriers as the most significant. Significant efforts need to be undertaken by firms to accomplished circular economy. Also circular economy regulation should be improved to make it easier for companies to implement strategies that will make them more sustainable.

## 1. Introduction

The current linear economy is based on converting natural resources into waste via production. This traditional model, in which goods are manufactured and then discarded as waste, deteriorates the environment. Although recycling is fully developed in our society, and improving resource efficiency is encouraged, activities focused on achieving this efficiency fail to consider the finite nature of material stock [1]. Conversely, a circular economy (CE), restores any damage done during resource acquisition while ensuring not much waste is generated during the product life cycle. Some authors state that CE may benefit or at least reduce waste or damage to the environment, economy and society [2] even that may not have any net effect on the environment [3]. This is why the CE is currently attracting the attention of the academic literature and institutions. The European Commission (EC) has also warned organizations and society of their important mission to pave the way for a new economic model [4]. In the EC communication titled *Closing the loop—An EU action plan for the Circular Economy* [5], a CE was defined as ‘one in which the value of products, materials and resources is maintained for as long as possible’, thus minimizing waste and resource use. This is a starting point, but increasing waste prevention, reuse, recycling and recovery are fundamental actions of both the action plan and the legislative package on waste [4].

From an academic viewpoint, the number of CE-related publications in top journals has increased rapidly since 2007, and most were published in the 2014–2016 period [6]. The need to clarify the concept of CE—and its goals, means and how to implement it—is incipient in the academic literature since the concept is novel [6,7,8,9,10]. However, it is generally accepted that the CE is a paradigm shift attempting to integrate economic activity and environmental wellbeing [7]; replace the end-of-life concept with the 4Rs -reducing, reusing, recycling and recovering- in production and consumption processes [9]; and slow down, close and narrow material and power loops [9,11]. Applied to the meso level, this means turning goods that are at the end of their service life into resources for others, thus stretching the economic life of goods and materials, closing loops and minimizing waste, that is, the CE replaces production with sufficiency [12,13]. 

The principal aims of implementing a CE strategy in an organization are to reduce virgin materials and waste output [14] and to protect the environment and prevent pollution [15]. In other words, a CE strategy is implemented to accomplish sustainable development through increased resource efficiency. The concept of sustainable development explains environmental quality, economic development and social equity [9], while protecting the environment and preventing pollution [14]. Nevertheless, sustainable development refers to a ‘development that meets the needs of the present without compromising the ability of future generations to meet their own needs’ [16] (p. 43). This definition underpins the assumption that resources are finite and have to be managed to sustain future generations [3].

Based on the document presented by the Ellen MacArthur Foundation [1], the CE rests on three principles: (1) preserving and enhancing natural capital by controlling finite stocks and balancing renewable resource flows; (2) optimizing resource yields by circulating products, components and materials in use at the highest utility; (3) fostering system effectiveness by revealing and designing out negative externalities. The 4Rs the CE is based on—reduction, reuse, recycling and recovery—are extracted from these principles. The third in the list, recycling, has been implemented within the traditional linear economy system—based on extract–produce–use–dump—because many policies have promoted it [6]. By increasing product longevity through better manufacturing and maintenance, the replacement rate decreases, resulting in reduced resource use [3]. Although recycling has been fully developed, it is still the tip of the iceberg. The CE will require changes in legislation, the way society produces and consumes innovations, while also using nature as an inspiration to respond to societal and environmental needs [7]. 

Considering resource availability is important when talking about implementing CE activities. Large enterprises are known to have more margins to invest in new production methods and can, therefore, implement these kinds of activities. Nevertheless, as the Organisation for Economic Co-operation and Development (OECD) stated [17], 95% of companies in OECD member countries are small and medium-sized enterprises (SMEs) and, as [18] mentioned, 99% of companies in the EU are SMEs. That is why the study of the CE strategy should focus on this kind of firm. 

The objective of this work is to study CE practices and analyze in depth CE behavior at a meso level. The novelty of the concept, the need for a literature background focused on business management, the lack of consensus on practices associated with a CE strategy and the shortage of empirical studies analyzing CE barriers are the motivation for this work. The paper is organized as follows. The following section reviews the theoretical framework that could explain the CE. The third section reviews the previous CE literature and highlights the need for further study. The fourth section defines the empirical study and presents the results. The fifth section contains the study’s conclusions. 

## 2. Previous Circular Economy Studies 

Since the concept of the CE is still emerging, there are few studies where CE drivers and barriers are analyzed, and most of them are based on reviews, merging CE literature with eco-innovation and sustainable development concepts. Some studies confirm that the CE has been promoted mainly by practitioners, the business community and policy makers, and interest in academic studies is now growing, thus making the CE a trending concept [9]. This may be the reason why there is currently no comprehensive and systematic analysis to understand the CE and, therefore, the emerging literature has concentrated on the limitations and characterization of the CE concept, trying to arrive at a consensus in the environmental management literature [6,7,8,9]. These studies have focused on finding a generally accepted definition of the CE by analyzing previous studies related to implementing this kind of activity.

Another group of studies has concentrated on developing a theoretical background essentially based on the industrial ecology perspective [3,19,20,21]. One of the conclusions these papers share is that industrial ecology tools (material power and water flows from industrial symbiosis) are needed to fully support the CE. There is also a need to develop a theoretical framework based on business management since research into the CE has paid particular attention to waste generation, resource use and environmental impact, while neglecting business and economic perspectives [21]. One of this study’s conclusions is that companies should not prioritize either environmental or economic benefits because the ultimate objective of CE implementation strategy is achieving a fully regenerative economy and natural environment. 

Empirical studies related to CE practices are scarce. In one of these works, Urbinati et al. [19] established four different modes of adopting CE principles in firms considering the value network and customer value proposition and interface: linear, downstream circular, upstream circular, and full circular, depending on the degree of circularity. Based on the case studies of 24 firms, these authors observed that full circular companies could be either large firms with more years of activity, or new ventures created to exploit the potential of circular business models. They also pointed out the need for future empirical research to analyze CE policies and objectives and create awareness of the need for product design practices. 

De-Jesus and Mendoça [22] analyzed the factors that influence implementing CE activities or policies using academic and ‘grey literature’. These authors used 40 works published in the 2006–2015 period to group CE drivers and barriers from hard to soft. They identified hard drivers and barriers with technical factors (such as the availability of technology, technical support, training, and so on) and economic factors (for example, capital requirements or transaction costs), and soft drivers and barriers with social, regulatory and institutional factors. They found that the CE is driven particularly by soft factors, and demonstrated the crucial role of institutional framing and increasing social awareness. They concluded that, even when CE practices are technically feasible, their implementation is often limited by economic and market limitations, thus underscoring the role of environmental innovation, considered an essential pathway for overcoming CE barriers. 

Ranta et al. [23] used a qualitative six-case study to examine the institutional CE drivers and barriers in China, the US and Europe. In their work, they stressed the lack of institutional support for other CE principles outside recycling, especially regulation-wise. They also found a major cultural cognitive barrier to reuse, which is the customer preference for new products, concluding that the general barrier to the CE could be the emphasis on recycling, which resonates with a lack of institutional support for reuse. 

Ormazabal et al. [18] conducted a survey study on SMEs in Navarre and the Basque Country in Spain and discovered that the most critical barriers are the lack of support from public organizations, insufficient financial resources and lack of customer interest in the environment—this idea is shared by Kirchherr et al. [24]. By conducting a factor analysis, the authors identified and named two different components for barriers in SMEs: hard barriers—lack of financial support, insufficient information management systems, lack of adequate technology, insufficient technical resources, insufficient financial resources, and lack of support from public institutions—and human-based barriers—lack of customer interest in the environment, lack of qualified personnel in environmental management, and commitment of the organization’s leaders. This work tried to shed light on CE literature by identifying the opportunities and barriers to implementing the CE in SMEs. The authors concluded that considering the activity sector while analyzing CE implementation is important because some industries are more willing to implement environmental strategies in some CE cycle phases. They also point out SMEs’ limited resources, short-term vision and lack of time in their everyday activities, which imply they do not see the CE as one of their priorities. 

As has been demonstrated, most of the literature on the CE focuses on reviewing the concept and trying to establish a generally accepted definition and practices that characterize CE activities. Few studies have analyzed the implementation of CE activities in business and the barriers companies have to overcome and, in those cases, the methodology is limited. The sample of this type of study is based on previous works, literature or samples focusing on limited geographical areas. In the following section a theoretical background is reviewed focusing on the industrial ecology, which is the most important theoretical view for CE at the time, and the resource based view.

## 3. Theoretical Framework 

The current industrial economy is known as a linear resource consumption model that follows a ‘take-make-dispose’ pattern. Following this linear model, firms in different industries use natural resources to generate products and sell them to customers, who then discards them as waste [25]. The traditional linear model assumes an unlimited supply of natural free of charge resources and an unlimited capacity of the environment to absorb waste and pollution [3,26]. Although great strides have been made in increasing resource efficiency—especially with the recycling policy—this model incorporates several waste and pollution sources along the supply chain [3,19]. These traditional linear consumption patterns—together with the tendency for the world’s population to grow and the exponential increase in the demand for raw materials, water and power—are limiting the availability of resources. As a result, we can observe the overuse of resources, the removal of natural resources from the environment and the reduction in the value of natural capital, what causes higher price levels and more volatility in many markets [25]. 

A CE is a regenerative industrial system by intention and design [1] which offers a new perspective on waste and resources management [27]. The concepts of restoration or regeneration are highly important in CE because they show that the industry itself aims to repair previous damage by developing new and better systems [3,28]. CE replaces the ‘end-of-life’ concept with restoration, promote the use of renewable energy, eliminates the use of toxic chemicals, which impair reuse, and aims to eliminate waste through the superior design of materials, products, systems and business models [1]. As Murray et al. [3] argued, the CE focuses on optimizing systems rather than components, and on achieving value from redesigning manufacture and service supply systems rather than simply improving resource utilization. Keeping this in mind, and focusing on firms’ circular behavior, there are some literature approaches that could be applied to the study of the transition from a linear to a circular economy. 

The resource based view has been used in academic studies to examine the internal needs to implement new or innovative practices for an organization [29]. This view proposes that internal needs include tangible and intangible resources and capabilities to reach a competitive advantage [30]. According to the natural resource-based view—the first step towards incorporating the challenge of the natural environment into strategic management—competitive advantage lies in the existence of internal resources and competencies that are valuable, rare and difficult to imitate, considering that (natural) resources are scarce [31,32,33,34]. The scarcity of these resources makes companies look for substitute resources, which enables them to create additional value. Besides the changing environment, all this requires major internal changes and cross-functional capabilities based on tacit competencies that allow firms to adapt to new scenarios. The literature in this respect demonstrates that *developing dynamic capabilities can be viewed as a learning process that contributes to building, exploiting and transforming new knowledge to address change* [35]. Dynamic capabilities are seen as a process related to companies’ ability to reconfigure the source of their resources to respond more efficiently to changes and create value [35,36]. This approach could be applied to the CE following the four principles of circular value creation contained in the Ellen Macarthur Foundation communication [25]: (a) the tighter the circles are, the larger the savings should be in the embedded costs in terms of resources; (b) keeping resources—such as products, components, and materials- in use longer; (c) the arbitrage value creation potential is rooted in the lower marginal costs of reusing the cascading (consecutive uses) material as a substitute for virgin material inflows and their embedded costs; (d) to generate maximum value, each of the above principles—requires a certain purity of material and quality of products and components. All these CE bases for value creation require the development of dynamic capabilities so that the firm can adapt to changes in the environment and benefit from the advantages of the value creation consequences of implementing CE activities. From this perspective, as Kabongo and Boiral [34] argued, firms are likely to develop dynamic capabilities for the CE as a result of a continued learning process. 

The CE could also be analyzed through an industrial ecology perspective, aimed at understanding the circulation of materials and whose holistic goal is to guide the transformation of the industrial system to a sustainable one [13,20]. Generally speaking, industrial ecology is the *means whereby humanity can deliberately approach and maintain sustainability, given continued economic, cultural and technological evolution* [21]. Specifically, the industrial ecology could be seen as a system view where the general objectives are to optimize the total materials cycle from virgin material, to finished material, to component, to product, to obsolete product, and to ultimate disposal [37,38,39]. Focusing on management, industrial ecology is the study of material flows through industrial systems and it aims to create closed-loop processes in which waste serves as an input, thus eliminating the notion of an undesirable by-product within and outside the industrial system [21,25]. Industrial ecology adopts a systemic viewpoint by developing production processes based on local ecological constraints, looking at their global impact from the outset, and attempting to shape them [25]. Industrial ecology could be applied at three levels [21,30]: (a) the factory or company level, where attention is paid to cleaner production; (b) inter-firm level, where collaboration and synergies are emphasized and industrial symbiosis (based on the biological analogy in nature: nutrients are cycled and power is cascaded down among the actors in the systems in a mutually beneficial manner [3,27]) could be achieved due to geographic proximity; (c) regional or global level. With the industrial ecology perspective, independent companies would create physical links to use each other’s waste as resources—by exchanging power, materials, water and by-products—and to slow down use cycles to delay waste output [3,20]. Focusing on the company level—meso level-, CE activities would be implemented along the entire value chain, creating multiple closed loops that make it easy to take advantage of reusing resource waste.

With this, the academic work analyzing the CE could be summarized in three general groups: (1) First, there is an important group of papers which objective is to work on a definition of CE that could be generally accepted [6,7,8,9]; (2) Second, there is a scarce number of academic works related to CE to the theoretical background of the concept [3,19,20,21]. Usually, these works are focused on industrial ecology background, and few of them focus on a management view; (3) The third group of papers is the one that encompasses empirical analysis [18,19,22,23]. Following this study’s objective, which is to analyze the status quo of the CE strategy and identify the typologies of CE activity implementation in firms, we focus on the natural resource-based view and the industrial ecology perspective to reach this objective. This allows us to discover which CE activities are most implemented, based on activity sector, following the recommendation of Ormazabal et al. [18], and the barriers that have to be overcome to implement this type of practice. All these is detailed in the next empirical section.

## 4. Empirical Study

### 4.1. Sample

To reach the objective proposed in this study we used the European SMEs and the Circular Economy database, which is based on Flash Eurobarometer Survey number 441 [40]. The survey was commissioned by the European Commission to explore SMEs circular economy activities following the ambitious circular economy package which was adopted in December 2015 [5]. The survey was carried out by TNS Political & Social network in the 28 Member States of the European Union between the 18th and 27th of April 2016. Therefore, this database takes information from the year 2015 on CE activities implemented in companies in European countries, as well as the barriers to these CE practices. Thus, our sample consists of 10,618 companies in 28 EU countries. The sample focuses on SMEs, as shown in Table 1. 

More than half the sample (62.97%) is composed of microenterprises with fewer than 10 employees, and there are 1456 cases with between 50 and 250 members of staff, representing 13.71% of the sample. It is also important to observe the sample companies’ business sector because their industry can limit CE activity implementation. Based on the Statistical Classification of Economic Activities in the European Community (NACE), more than 30% of the sample belongs to retail trade and transportation. Major sectors, such as manufacturing (13.63%), construction (11.50%), and scientific and technical activities (12.82%) are also represented in the sample.

### 4.2. CE Activity Implementation and Barriers

In Flash Eurobarometer Survey number 441 we found five different internal measures for the CE: (1) re-planning of the way water is used to minimize usage and maximize re-usage; (2) use of renewable energy; (3) re-planning energy usage to minimize consumption; (4) minimizing waste by recycling and reusing waste or selling it to another company; (5) redesigning products and services to minimize the use of materials or use recycled materials. Firms were asked if they had performed any of these activities in the last three years (2013–2015). Out of the 10,618 total answers, 7843 said they had implemented or were implementing at least one of the CE measures—we call these companies in-going firms—while 2775 said they had not implemented any CE activities—no-going firms. The survey first asked in-going firms about the issues they had encountered when undertaking CE activities. Five barriers were listed: (1) lack of human resources; (2) lack of expertise to implement these activities; (3) complex administrative or legal procedures; (4) cost of meeting regulations or standards; (5) difficulties in accessing finance. No-going firms were asked about the reasons why they had not performed any CE-related activity. The possible reasons were: (1) lack of human resources; (2) lack of expertise to implement these activities; (3) no clear idea about cost benefits or improved work processes; (4) no clear idea about investment required; (5) complex administrative or legal procedures; (6) cost of meeting regulations or standards; (7) difficulties in accessing finance. Table 2 shows that the CE activity most performed by more than 56% of in-going firms is recycling and reusing, followed by minimizing power consumption by at least 40% of the firms. In-going firms are also characterized by meeting complex legal procedures and regulation standards while implementing CE activities. No-going firms, however, stand out for financial barriers, such as no clear idea about cost benefits or the investment required (Table 2). 

The information in Table 3 shows the differences between activity sectors in the in-going and no-going companies. Using a chi-square test we examined whether there are statistically significant differences in the distribution of no-going and in-going enterprises. 

**Figure 1:**
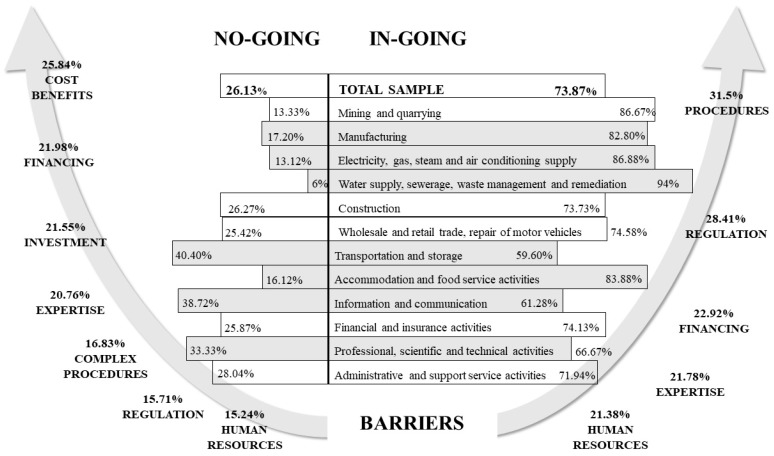
Circular economy implementation and barriers.

A high number of in-going firms work in the manufacturing, electricity, construction and accommodation and food service sectors. These activity sectors seem to be more proactive in implementing CE practices in their processes. Other sectors like transport, information and communication, and professional, scientific and technical activities stand out for not implementing any CE practices (no-going companies). All this information is detailed in Figure 1, which summarizes the information contained in Table 1, Table 2 and Table 3. 

In the right part of Figure 1, we can observe in-going firms for every activity sector; the darkest are those sectors with more in-going firms than expected (statistically significant differences). Conversely, no-going firms are on the left side, and we have also shaded where there are more no-going firms than expected. The other activity sectors that are not shaded behave in the same manner as the mean of the entire sample. Finally, in Figure 1 we find the barriers that in-going companies found compared with no-going ones. For example, lack of human resources seems to be the same in both groups, with a low score. Nevertheless, there seem to be some differences in the perception of financing, procedure and regulation barriers. While no-going firms did not think regulations were an important barrier, with 15.24%, nearly 30% of the in-going companies stated they were the second most important obstacle to implementing CE activities. We found the same situation with complex administrative or legal procedures. It seems that in-going firms find more barriers related to regulations, standards and procedures, while no-going firms find barriers related to investment and searching for financing. Nevertheless, this is an anticipated conclusion and more research has to be conducted in this area.

### 4.3. CE Behavior for in-Going Firms

**Figure 2:**
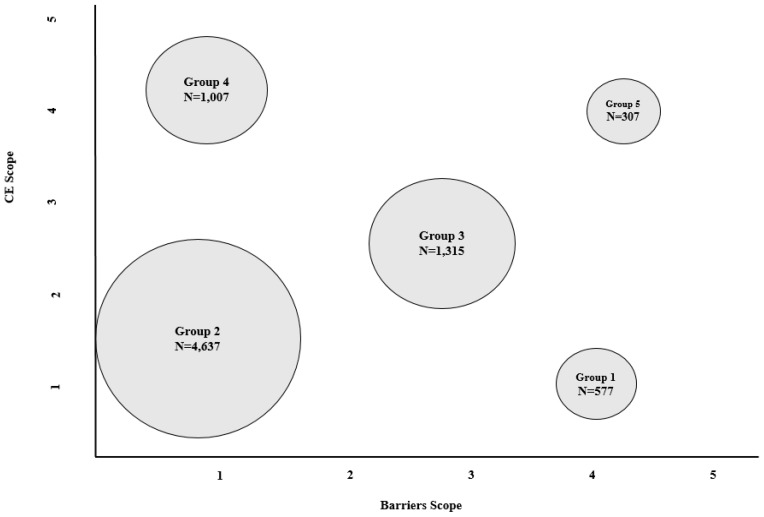
CE Scope and Barriers to CE Scope.

With the objective of analyzing CE behavior in depth, two variables were built on the total number of CE activities and barriers: CE scope and barriers scope. Firstly, we constructed the CE scope variable using the total number of implemented CE practices. This variable is the sum of implemented CE activities, so it has a range of 1 to 5 (in-going firms should have implemented at least one CE activity). Then, for the construction of the barriers scope variable we also totaled the number of barriers to implementing CE activities, resulting in a variable range of 0 to 5. With these two variables, we used the cluster analysis technique to obtain a typology of CE behavior. Figure 2 shows that we obtained five groups for the CE scope and barriers scope in the cluster analysis. 

There seems to be five differentiated behaviors concerning CE activity implementation, ranging from firms that do not have much CE scope and barriers scope, to those that, on average, have an integrated CE strategy and have overcome many barriers. The first group contains 577 companies that, on average, have implemented 1.49 CE activities and found 4.16 barriers. These companies find too many barriers for the small amount of CE-related activities they implement. The second group is the largest, with 4637 companies; this seems to be the most common CE behavior, with 1.77 CE activities implemented and 0.43 barriers on average. The third group comprises 1315 firms; companies in this group seem to implement more CE practices (2.46 in average) and also overcome more barriers, with an average of 2.68. The fourth group seems to contain the most efficient companies for CE activity implementation; this group has 1007 companies that, on average, have implemented 4.25 CE activities and they found fewer barriers to it (0.8). The fifth and last, the smallest group, also contains very proactive CE-implementing companies with an average of 4.29 activities, and they found 3.76 barriers. The behavior followed by group number four, with a high scope in implementing the CE and low barriers, seems to be the most efficient. However, in all cases, we observed that the CE behavior seems to follow a reactive-proactive pattern.

We developed a variance analysis (ANOVA) means contrast for the five groups obtained in the cluster analysis and the CE activities and barriers to analyze this reactive-proactive pattern in depth. As shown in Table 4, there are statistically significant differences between all CE activity and barrier groups. Certain processes appeared in the different groups relating to implementing CE activities. We observed that the first CE activity implemented in companies seems to be recycling/reuse, followed by minimizing power consumption and product redesign. We also noted that groups 4 and 5 implemented all the CE activities above the sample’s mean. These two groups differ in the barriers overcome: group four highlighted the complex administrative or legal procedures as an issue, while group number five highlighted all of them, paying special attention to the cost of meeting regulations or standards.

There seems to be a progressive process in the implementation of CE activities, due to the behavior of the different groups. It was noted that all the groups have implemented recycling and reuse. As mentioned in the theoretical background, this practice is ingrained in society and industry. The second most implemented activity by in-going firms is related to minimizing power consumption, followed by product redesign to minimize the use of materials or use recycled material. After implementing this practice, it seems firms have reached a scale economy, where implementing one more practice is not an extra effort. We observed that groups four and five have achieved this point, and they also rethink the way water is used and follow renewable energy practices. Not only does there appear to be a pattern from reactive to proactive behavior in implementing CE activities, but also a progressive switch from control to prevention activities. 

We also observed barrier typology. One barrier appeared in all the groups: administrative or legal procedures. This barrier is followed by the cost of meeting regulations and standards and then the lack of human resources. After coming up against and/or overcoming these three issues, firms also seem to find it difficult to access the finance and expertise needed to implement CE activities.

After demonstrating the apparent typology of CE behavior, it is interesting to describe how each group is obtained and behaves. Table 5 shows the statistically significant differences between size, R&D investment, activity sector and country in the distribution of each CE group. Firms in group one, which stand out for implementing recycling or reuse and coming across all the CE barriers, are, on average, microenterprises with less than 5% of R&D investment. Apparently, they concentrate on manufacturing, construction, retail and transportation activities and are located in eastern European countries (Hungary, Latvia, Poland, Slovakia and Romania). Group two is characterized by a low scope in CE activities (only implementing recycling and minimizing power consumption) and low barrier scope (only administrative and legal procedures). Firms in this group are also microenterprises, but they invest a little more in R&D; they belong to the information and communication and scientific and technical sectors—which leads us to believe that the increase in R&D investment could be due to their business type—and are mostly located in Germany, Italy and Denmark. Group three, with a medium scope for CE activities and barriers, is slightly larger than the two previous groups. These companies also invest more in R&D than those in groups one and two, almost 15% of their turnover on average, and they belong to the construction and retail sectors; French companies are predominant in this group. Finally, groups four and five stand out for implementing all the CE-related activities. Firms in these groups are larger and also invest more in R&D. Both groups find that legal procedures, regulations and human resource standards are barriers. They also share the same sectors, namely manufacturing and water supply and waste management, and country of location (Ireland). This could suggest that these sectors and this country could have more procedures or costs that hinder the implementation of CE activities.

## 5. Conclusions

This work has found that the CE concept is novel and emerging, above all in the academic literature on management. As public and private institutions have stated [1,4], there is now a need to change the system and stop using linear production models. The importance of SMEs in Europe highlights the need to analyze the CE in terms of these firms. Changing the system, paradigm and industrial model requires a series of small steps to gradually encourage circular production processes, thus promoting the implementation of the 4Rs. 

This study has confirmed the need for a theoretical framework in environmental management literature and it has attempted to provide an overview of the CE with a focus on resources and dynamic capabilities, but without forgetting industrial ecology. These two theoretical perspectives should be used jointly to analyze the CE model as they complement each other. While the industrial ecology or science of sustainability introduces natural biological cycles into business management, turning the waste of one process into the raw material of another, the dynamic capabilities perspective views the introduction of new circular measures as the creation of knowledge through a process of adapting to an environment that is constantly changing. When implementing CE activities in a business, the company should consider its dynamic environment, and stakeholders’ movements, positions and preferences. From the perspective of dynamic capabilities, CE activity implementation is viewed as a process whereby companies develop skills that will enable them to take advantage of their efforts and attain economies of scale in introducing sustainability measures. 

Our review of the literature has uncovered that the few studies on the CE analyze it from a concept perspective, seeking its taxonomy and exploring the characteristics of this type of activity. Not many studies focus on the empirical analysis of this type of strategy. In our study, we have analyzed the behavior of several companies to obtain an implementation typology of these practices. We have observed that the CE behavior is a gradual process that starts by implementing material recycling and reuse measures. The next step is to put into practice measures to minimize power consumption and to redesign products. As a last step, the most proactive firms in implementing CE measures also rethink their water use and turn to renewable energy. We observed that CE measures are implemented gradually, starting with activities involving control measures and ending with putting preventive practices in place. These implementation profiles, ranging from the most reactive behavior, based on introducing pollution control measures, to the most proactive with prevention measures, coincide with previous studies analyzing the implementation of proactive environmental strategies in business [41,42,43]. 

Implementing CE practices could be conditioned by several factors. Our work has analyzed the barriers affecting both proactive CE companies, called in-going firms, and reactive companies, called no-going firms. We discovered that in-going firms believe they have overcome different barriers to no-going firms. Firms that have not implemented CE measures view financing, investment and cost–benefit barriers as the most significant. In other words, companies that do not implement CE measures generally seem to believe that the factors that have prevented them from doing so are essentially economic. 

In the cluster analysis classifying the in-going companies based on the CE measures they have implemented and the barriers they came across, we have observed five typologies whose scope differs, in both the number of CE measures and the number of barriers. After obtaining the typologies, we found that the most proactive companies in implementing CE measures generally come across certain common barriers: administrative processes, regulations and a lack of human resources to perform these practices. These results partly coincide with previous authors’, such as Urbinati et al. [19] and Ranta el al. [23], who refer to the need for institutional support, for example policies and regulations that enable CE measures to be created and implemented in companies. They do not coincide with the results obtained by Ormazabal et al. [18], who concluded that the lack of financial resources was one of the main CE barriers. The results also show that firms implementing the most measures are medium-sized. There also seems to be a positive relationship between the scope of CE activities and R&D investment. In view of the results, considering external factors, such as the company’s business sector or country location, is also important. Regulations, standards or practices can determine how or whether businesses will implement CE measures and react to any barriers they come across. Consequently, we can conclude that CE regulations should be improved to make it easier for companies to implement strategies that will make them more sustainable. 

It is important to note that the concept of barrier should not be seen as negative. When companies overcome barriers, it implies they have acquired and accumulated knowledge and this helps them to implement practices better, have more understanding of the production process and identify possible improvements, and, therefore, create more circular, sustainable and efficient processes. 

The main limitation of this work is that these are initial results. Due to the lack of data for analysis and the fact that the CE is a recent concept, the factors promoting the implementation of these practices and the effects they have on the companies performing them should be explored in more depth. As a result, we propose the factors that facilitate the CE and its impacts on firms as a future line of research. 

## Figures and Tables

**Table 1:** Sample Distribution.

	*N*	%
Size		
1–9 employees	6687	62.97
10–49 employees	2475	23.30
50–250 employees	1456	13.71
**Activity Sector**		
Mining and quarrying	30	0.28
Manufacturing	1448	13.63
Electricity, gas, steam and air conditioning supply	61	0.57
Water supply, sewerage, waste management and remediation	100	0.94
Construction	1222	11.50
Wholesale and retail trade, repair of motor vehicles and	3627	34.15
Transportation and storage	656	6.17
Accommodation and food service activities	757	7.12
Information and communication	483	4.54
Financial and insurance activities	348	3.27
Professional, scientific and technical activities	1362	12.82
Administrative and support service activities	524	4.93
**Country**		
France	401	3.8
Belgium	401	3.8
The Netherlands	403	3.8
Germany	400	3.8
Italy	400	3.8
Luxembourg	200	1.9
Denmark	402	3.8
Ireland	400	3.8
United Kingdom	400	3.8
Greece	400	3.8
Spain	400	3.8
Portugal	400	3.8
Finland	401	3.8
Sweden	400	3.8
Austria	400	3.8
Cyprus (Republic)	201	1.9
Czech Republic	400	3.8
Estonia	400	3.8
Hungary	402	3.8
Latvia	402	3.8
Lithuania	400	3.8
Malta	200	1.9
Poland	401	3.8
Slovakia	400	3.8
Slovenia	403	3.8
Bulgaria	400	3.8
Romania	401	3.8
Croatia	400	3.8
	10,618	100

**Table 2:** In-Going and No-Going firm’s distribution.

	*N*	% ^(^*^)^
**In-Going Firms (*N* = 7843)**		
**CE Activities’ Implementation**		
Water	1998	25.47
Renewable Energy	1850	23.59
Energy Consumption	4323	55.12
Recycle/Reuse	6052	77.16
Redesign	3652	46.56
**Barriers to CE**		
Lack of human resources	1677	21.38
Lack of expertise to implement these activities	1708	21.78
Complex administrative or legal procedures	2459	31.35
Cost of meeting regulations or standards	2228	28.41
Difficulties in accessing finance	1798	22.92
**No-Going Firms (*N* = 2775)**		
**Barriers to CE**		
Lack of human resources	423	15.24
Lack of expertise to implement these activities	576	20.76
No clear idea about cost benefits or improved work	717	25.84
No clear idea about investment required	598	21.55
Complex administrative or legal procedures	467	16.83
Cost of meeting regulations or standards	436	15.71
Difficulties in accessing finance	610	21.98

^(^*^)^∑≠100%; The percentage is calculated with *N* = 7843/2775.

**Table 3:** Sample distribution. In-Going and No-Going firms.

	No-Going			In-Going				Total
	*N*	%	Barriers Scope	*N*	%	CE Scope	Barriers Scope	
Mining and quarrying	4	13.33	0.50	26	86.67	2.10	1.38	30
Manufacturing	249	17.20 ***	1.56	1199	82.80 ***	2.01	1.38	1448
Electricity, gas, steam and air conditioning supply	8	13.11 ***	0.50	53	86.89 ***	2.52	1.26	61
Water supply, sewerage, waste management and remediation	6	6.00 ***	1.33	94	94.00 ***	2.58	1.69	100
Construction	321	26.27	1.72	901	73.73	1.72	1.53	1222
Wholesale and retail trade, repair of motor vehicles and	922	25.42	1.44	2705	74.58	1.62	1.20	3627
Transportation and storage	265	40.40 ***	1.21	391	59.60 ***	1.27	1.32	656
Accommodation and food service	122	16.12 ***	2.13	635	83.88 ***	2.18	1.49	757
Information and communication	187	38.72 ***	1.10	296	61.28 ***	1.21	1.04	483
Financial and insurance activities	90	25.86	1.22	258	74.14	1.68	0.96	348
Professional, scientific and technical	454	33.33 ***	1.04	908	66.67 ***	1.44	1.00	1362
Administrative and support service	147	28.05	1.15	377	71.95	1.68	1.06	524
Total	2775	26.13	1.38	7843	73.87	1.68	1.26	10,618

Chi-square test. The difference between observed and expected values is statistically significant *** *p*-value < 0.01.

**Table 4:** CE behavior and barriers.

	$\bar{x}$	${\bar{x}}_{1}$	${\bar{x}}_{2}$	${\bar{x}}_{3}$	${\bar{x}}_{4}$	${\bar{x}}_{5}$	ANOVA	Duncan Test
**CE activities implemented**								
Water	0.18	0.13	0.13	0.26	**0.71**	**0.77**	640.80	${\bar{x}}_{1}$= ${\bar{x}}_{2}$
Renewable Energy	0.17	0.07	0.14	0.20	**0.68**	**0.68**	585.26	${\bar{x}}_{4}={\bar{x}}_{5}$
Redesign	0.34	0.30	0.34	**0.55**	**0.91**	**0.91**	443.04	${\bar{x}}_{1}={\bar{x}}_{2};\;{\bar{x}}_{4}={\bar{x}}_{5}$
Energy Consumption	0.40	0.32	**0.44**	**0.61**	**0.97**	**0.96**	389.87	${\bar{x}}_{4}={\bar{x}}_{5}$
Recycle/Reuse	0.57	**0.66**	**0.71**	**0.82**	**0.97**	**0.97**	118.08	${\bar{x}}_{4}={\bar{x}}_{5}$
**CE scope**	1.68	1.49	1.77	2.46	4.25	4.29	3,465.75	${\bar{x}}_{4}={\bar{x}}_{5}$
Lack of expertise to implement these activities	0.21	**0.82**	0.07	0.46	0.10	**0.63**	976.23	${\bar{x}}_{2}={\bar{x}}_{4}$
Difficulties in accessing finance	0.22	**0.80**	0.08	0.47	0.11	**0.74**	972.42	${\bar{x}}_{2}={\bar{x}}_{4}$
Lack of human resources	0.21	**0.77**	0.07	0.43	**0.12**	**0.64**	836.27	
Cost of meeting regulations or standards	0.28	**0.88**	0.09	**0.63**	**0.20**	**0.88**	1368.77	${\bar{x}}_{1}={\bar{x}}_{5}$
Complex administrative or legal procedures	0.31	**0.90**	**0.11**	**0.69**	**0.26**	**0.86**	1280.48	${\bar{x}}_{1}={\bar{x}}_{5}$
**Barriers scope**	1.26	4.16	0.43	2.68	0.80	4.16	6619.53	

ANOVA: Reject H0: “${\bar{x}}_{1}={\bar{x}}_{2}={\bar{x}}_{3}={\bar{x}}_{4}={\bar{x}}_{5}$” for *p*-value < 0.000; and Duncan Test: Reject H0: “${\bar{x}}_{i}={\bar{x}}_{j}$”, for all *i* ≠ *j*. In bold and shadowed the CE activities and CE barriers according to the intensity and scope group where belong (*p*-value < 0.00).

**Table 5:** In-Going typology.

		${\bar{x}}_{1}$		${\bar{x}}_{2}$		${\bar{x}}_{3}$		${\bar{x}}_{4}$		${\bar{x}}_{5}$	
**Size**											
Micro (1–9 Employees)	59.3	**357**	**61.872**	**2905**	**62.648**	721	54.829	504	50.050	166	54.072
Small (10–50 Employees)	24.7	146	25.303	1096	23.636	**358**	**27.224**	**254**	**25.223**	87	28.339
Medium (50–250 Employees)	15.9	74	12.825	636	13.716	236	17.947	**249**	**24.727**	**54**	**17.590**
**% of R&D**											
Less than 5% of turnover	71.9	425	76.715	**3527**	**82.119**	894	71.406	606	68.090	190	66.667
Between 5–10%	9.0	52	9.386	**331**	**7.707**	**167**	**13.339**	**114**	**12.809**	**39**	**13.684**
Between 10–15%	5.3	31	5.596	192	4.470	**86**	**6.869**	**77**	**8.652**	**27**	**9.474**
More than 15% of turnover	6.6	46	8.303	245	5.704	105	8.387	**93**	**10.449**	**29**	**10.175**
**Activity sector**											
Mining and quarrying	0.3	2	0.347	14	0.302	5	0.380	4	0.397	1	0.326
Manufacturing	15.3	87	15.078	625	13.479	**235**	**17.871**	**191**	**18.967**	**61**	**19.870**
Electricity, gas, steam and air conditioning supply	0.7	1	0.173	26	0.561	8	0.608	**15**	**1.490**	3	0.977
Water supply, sewerage, waste management and remediation	1.2	5	0.867	36	0.776	**23**	**1.749**	**21**	**2.085**	**9**	**2.932**
Construction	11.5	**86**	**14.905**	477	10.287	**187**	**14.221**	103	10.228	**48**	**15.635**
Wholesale and retail trade, repair of motor vehicles and	34.5	212	36.742	**1704**	**36.748**	426	32.395	278	27.607	85	27.687
Transportation and storage	5.0	36	6.239	234	5.046	66	5.019	40	3.972	15	4.886
Accommodation and food	8.1	54	9.359	313	6.750	105	7.985	118	11.718	**45**	**14.658**
Information and communication	3.8	20	3.466	**207**	**4.464**	38	2.890	26	2.582	5	1.629
Financial and insurance	3.3	9	1.560	157	3.386	45	3.422	43	4.270	4	1.303
Professional, scientific and technical	11.6	45	7.799	**609**	**13.133**	125	9.506	109	10.824	20	6.515
Administrative and support	4.8	20	3.466	235	5.068	52	3.954	59	5.859	11	3.583
Water supply, sewerage, waste management and remediation	0.3	2	0.347	14	0.302	5	0.380	4	0.397	1	0.326
**Country**											
France	3.9	**49**	**8.492**	123	2.653	**83**	**6.312**	31	3.078	**23**	**7.492**
Belgium	4.3	30	5.199	172	3.709	63	4.791	**58**	**5.760**	14	4.560
The Netherlands	4.0	17	2.946	178	3.839	65	4.943	45	4.469	11	3.583
Germany	4.0	11	1.906	**209**	**4.507**	43	3.270	44	4.369	10	3.257
Italy	3.6	21	3.640	**187**	**4.033**	40	3.042	26	2.582	10	3.257
Luxembourg	2.2	8	1.386	89	1.919	21	1.597	**45**	**4.469**	8	2.606
Denmark	3.6	5	0.867	**229**	**4.939**	20	1.521	22	2.185	3	0.977
Ireland	4.7	20	3.466	194	4.184	62	4.715	**66**	**6.554**	**23**	**7.492**
United Kingdom	4.4	17	2.946	220	4.744	43	3.270	**57**	**5.660**	12	3.909
Greece	3.7	20	3.466	183	3.947	37	2.814	36	3.575	11	3.583
Spain	4.3	27	4.679	189	4.076	60	4.563	45	4.469	20	6.515
Portugal	4.4	21	3.640	194	4.184	54	4.106	**64**	**6.356**	11	3.583
Finland	4.1	11	1.906	169	3.645	62	4.715	**64**	**6.356**	15	4.886
Sweden	3.9	12	2.080	192	4.141	44	3.346	43	4.270	12	3.909
Austria	4.4	13	2.253	192	4.141	61	4.639	**64**	**6.356**	13	4.235
Cyprus (Republic)	1.9	7	1.213	**106**	**2.286**	13	0.989	19	1.887	3	0.977
Czech Republic	3.8	22	3.813	172	3.709	**69**	**5.247**	20	1.986	13	4.235
Estonia	2.7	6	1.040	**160**	**3.451**	19	1.445	23	2.284	2	0.651
Hungary	3.3	**33**	**5.719**	128	2.760	**62**	**4.715**	24	2.383	15	4.886
Latvia	3.0	**36**	**6.239**	131	2.825	48	3.650	13	1.291	4	1.303
Lithuania	2.8	21	3.640	**148**	**3.192**	27	2.053	15	1.490	5	1.629
Malta	2.4	5	0.867	**127**	**2.739**	19	1.445	33	3.277	5	1.629
Poland	3.4	**48**	**8.319**	120	2.588	**81**	**6.160**	10	0.993	11	3.583
Slovakia	3.5	**29**	**5.026**	174	3.752	48	3.650	20	1.986	6	1.954
Slovenia	3.7	17	2.946	162	3.494	44	3.346	48	4.767	**19**	**6.189**
Bulgaria	2.5	17	2.946	**136**	**2.933**	33	2.510	8	0.794	4	1.303
Romania	3.4	**35**	**6.066**	126	2.717	55	4.183	31	3.078	16	5.212
Croatia	4.2	19	3.293	**227**	**4.895**	39	2.966	33	3.277	8	2.606

In bold and shadowed the in-going typology according to the group where belong (*p*-value < 0.00).
